# Clinical and biochemical effects of cryotherapy protocols on mandibular premolars with symptomatic apical periodontitis: a randomized clinical trial

**DOI:** 10.1007/s00784-026-07073-6

**Published:** 2026-08-22

**Authors:** Maram Farouk Obeid, Reem Mohammed Amr Sharaf, Ibraheem Mohamed Ibraheem Hamza

**Affiliations:** Department of Endodontics, Faculty of Dentistry, Ain Shams University, Cairo, Egypt

**Keywords:** Cryotherapy, Post-operative pain, Substance P, Cold irrigation, Randomized clinical trial, Symptomatic apical periodontitis, Pulpitis, Clinical outcomes

## Abstract

**Objectives:**

The objective of this study was to evaluate the effectiveness of different cryotherapy techniques in reducing postoperative pain (POP) and proinflammatory Substance P (SP) levels in patients with symptomatic apical periodontitis.

**Materials and methods:**

A randomized controlled trial was conducted on patients with mandibular premolars diagnosed with irreversible pulpitis and symptomatic apical periodontitis. Participants were assigned to either Group A (control), Group B (intraoral cryotherapy) using ice-gel packs, Group C (intra-canal cryotherapy with saline) with a final flush of cold saline at 2–4 °C for 10 min and Group D (intra-canal cryotherapy with hypochlorite) (NaOCl, 2–4 °C) during chemo-mechanical preparation. (POP) was measured using a visual analogue scale (VAS), and SP levels were assessed from apical fluid samples using enzyme-linked immunosorbent assay (ELISA).

**Results:**

Showed that Group D produced a statistically significant reduction in POP (*p* < 0.001), while Groups B and C showed intermediated POP reductions and the Group A consistently demonstrated the highest POP values. Regarding SP, Group C demonstrated the greatest decrease in SP levels and the largest pre- to post-treatment difference (*p* < 0.001). Group D achieved intermediate reductions, whereas Group B yielded outcomes comparable to the Group A. A moderate to strong positive correlation was observed between SP levels and VAS scores 6 h postoperatively. These findings confirm that cryotherapy techniques, particularly cold irrigants, are effective in reducing POP and SP expression.

**Conclusion:**

Different Cryotherapy techniques effectively reduce (POP) and modulate SP expression. Irrigation with cold hypochlorite throughout chemo mechanical process was most effective for pain reduction while final flush with cold saline achieved the greatest decrease in SP.

**Clinical relevance:**

It provides a straightforward approach to alleviating postoperative pain in patients with symptomatic apical periodontitis.

**Clinical Implication:**

Cryotherapy techniques should be more widely incorporated into endodontic clinical practice, as they effectively reduce POP and regulate SP levels. Also, it should be supported as valuable adjunct for improving patients’ outcomes. The trial was registered at ClinicalTrials.gov (NCT07138365, release date 22/8/2025).

## Introduction

In endodontic practice, (POP) management is crucial, this unpleasant state might be anticipated, particularly in teeth that exhibit symptomatic apical periodontitis, pulp necrosis, and preoperative discomfort [1]. Consequently, it is believed that controlling it is essential to maximize the effectiveness of the treatment [2]. Peri-radicular tissues are vulnerable to injury from a variety of mechanical, chemical, and microbial sources [3]. Additionally, the instruments and approaches employed in treatment highly effects the status of peri-radicular area [4]. Numerous techniques have been attempted to treat (POP), such as the use of corticosteroids and analgesics [2].

One technique to treat POP is form of cold therapy called cryotherapy, which has grown in popularity because of its ability to reduce muscular spasms, manage edema, and relieve pain [5]. It provides pain alleviation and lessens tissue damage via a series of biophysical actions [6]. This is achieved by minimizing oxygen consumption and cellular metabolic activity [6]. The production of free radicals, which fuel inflammation, is reduced by this method. Interestingly, a “hunting phenomenon” takes place when tissue temperature drops below a specific threshold, which is approximately 15 °C [7]. One proposed mechanism underlying cryotherapy’s analgesic effect involves an initial phase of vasoconstriction followed by subsequent vasodilation, which may contribute to reduced nociceptor activity and inflammation [7]. It also slows down nerve signal transmission. The myelinated A-delta fibers, which speed up the transmission of pain impulses, entirely stop working around 7 °C [7]. At 3 °C, however, the nonmyelinated C-fibers that provide delayed pain and temperature sensations cease to operate [7]. Additionally, cryotherapy causes vasoconstriction, which reduces swelling by narrowing blood vessels and preventing fluid from seeping into the tissues [7]. The temperature differential between the tissue and the administered therapy, the length of exposure, the properties of the cooling agent, and the features of the targeted tissue are some of the variables that affect how efficient cryotherapy is [7].

Substance P (SP) is one of these proinflammatory mediators that has been found to be a crucial neuropeptide in neurogenic inflammation and nociceptive transmission [8]. It is released from sensory nerve endings in response to tissue injury and inflammatory stimuli, where it promotes vasodilation, plasma extravasation, and recruitment of inflammatory cells [8]. Particularly in the tooth pulp, it causes edema and plasma extravasation, which alters permeability and causes vascular abnormalities [9]. An increase in tissue volume is the result of capillary dilatation and fluid transudation [10]. Pressure may increase as a result of swelling inside the tooth pulp, which is enclosed by hard calcified walls [8]. Since it activates the dental nerves, this increase in pressure is interpreted as pain [11]. Because of its high association with symptomatic pulpitis and its proven use as a trustworthy biochemical marker for neurogenic pain in earlier endodontic research, SP was chosen as the study’s focal point [9].

This study evaluates both postoperative pain (POP) and Substance P (SP) levels to provide a comprehensive assessment of cryotherapy’s effectiveness in endodontic treatment. By examining the relationship between biochemical markers and clinical symptoms, the study further explores the correlation between pain scores and SP levels in patients with symptomatic apical periodontitis undergoing cryotherapy. The techniques investigated include intraoral cryotherapy using gel packs, intracanal cryotherapy in the form of a final flush with cold saline, or irrigation with cold sodium hypochlorite (NaOCl) throughout the chemo-mechanical preparation process.

The null hypothesis was that the experimental groups receiving cryotherapy and the control group would not differ significantly in POP or SP levels.

## Materials and methods

### Study design and ethics declarations

This study employed a randomized, controlled clinical trial design, The study is approved by the Ethical Committee of Faculty of Dentistry Ain Shams University (FDASU) with Approval code: FDASU Rec IR052432. Also, the trial is registered at ClinicalTrials.gov with number NCT07138365 with initial release date 22/8/2025.

### Sample size calculation

The sample size calculation was performed using data from a previous study [12]. with G*Power version 4.5.0, by adopting an alpha (α) level of (0.05), a beta (β) level of (0.2) (power = 80%) and an effect size (d) of (0.697). Based on this analysis, the predicted sample size was determined to be a total of 40 cases, with 10 participants assigned to each group. Noted that, the calculated sample size is a minimum estimation. If it is found to be minor, it can be safely increased.

### Patients’ selection

Patients were selected from the normal group of people visiting the Dental Teaching Hospital at Ain Shams University. Participants required to be between the ages of 18 and 45, diagnosed with irreversible pulpitis and apical periodontitis, free of any underlying medical disorders, and free of analgesics for the week before the session.

According to radiographic analysis, eligible individuals had to have a mandibular single-rooted premolar identified. Furthermore, individuals reported > 7 VAS for both preoperative and percussion pain. Additionally, patients should have exhibited exaggerated prolonged lingering pain responses to cold application that was sustained for few minutes after removal of stimulus (Endo-Frost, Coltene-Whaledent, Switzerland).

Diagnosis was confirmed during access cavity preparation, evidenced by profuse pulpal bleeding. Exclusion criteria comprised pregnancy, anatomical variations in root canal morphology, and refusal to participate. Prior to enrollment, all patients were thoroughly informed about the study procedures and treatment protocol, and provided written informed consent.

### Patients’ randomization, allocation, and preparation

Eligible patients were randomly assigned to one of three comparative groups using computer-generated randomization. The allocation sequence was concealed by an independent assistant, who prepared sealed black envelopes containing group assignments. Prior to treatment initiation, each patient selected an envelope at random from a box, thereby determining their group allocation. The study employed a double-blind design, ensuring that both the participants and the outcome assessor remained unaware of the assigned interventions throughout the trial.

All patients received an inferior alveolar nerve block using Artinibsa (Inibsa Dental 4% with 1:100,000 epinephrine). In cases requiring supplemental anesthesia, intra-pulpal injection was administered with a pressure syringe. Access cavities were prepared under copious irrigation, and teeth were isolated using a rubber dam. Upon pulp exposure, profuse bleeding within the chamber confirmed the diagnosis.

The initial glide path was established with a #10 K-file (Mani, Inc., Japan), followed by coronal flaring using an M-Pro orifice opener rotary file (19 mm length, 8% taper; Foshan Stardent Equipment Co. Ltd., Guangdong, China). Working length was determined to be 0.5 mm short of the full canal length using an electronic apex locator (Root ZX; J. Morita Corp., Tokyo, Japan), and verified with intraoral periapical radiography.

The initial baseline apical fluid (AF) sample (S1) was collected at this stage. A sterile size 20 paper point with a 2% taper (DiaDent Group, Seoul, Korea) was inserted 2 mm beyond the root apex and left in place for 30 s. It was then transferred to Eppendorf tubes containing 0.5 mL of phosphate-buffered saline (pH 7.4), which were subsequently stored at 10 °C [6]. Mechanical canal preparation was performed using the M-Pro rotary file system (Foshan Stardent Equipment Co., China), comprising instruments #20/4%, #25/6%, #35/4%, and #40/4%.

### Patients’ classification and intervention

#### Group A Control group (*n* = 45)

The canals were irrigated with NaOCl at room temperature, followed by a final flush with saline at room temperature.

#### Broup B Intra-oral group (*n* = 45)

Intraoral cryotherapy was administered by placing a custom-made plastic pack (2.5 × 5 cm) filled with ice gel (DonJoy Orthopaedic Pty Ltd, Normanhurst, New South Wales, Australia) on the buccal vestibule directly over the treated tooth. Patients were instructed to retain the pack intraorally for 10 min, with the option to remove it for 2 min if they experienced excessive cold or a burning sensation [13]. Throughout the cryotherapy session, a thermocouple device was used to monitor and maintain the temperature at 10 °C. A designated member of the research team ensured strict adherence to the temperature protocol. If the temperature exceeded the target threshold, the gel pack was promptly replaced to restore the desired range. Each pack was applied for 10 min, with a minimum of three cycles completed over a 30-minute period, incorporating 2-minute breaks between applications. Following the completion of the three cryotherapy cycles, the second apical fluid sample (S2) was collected as previously described.

#### Group C Intra-canal cryotherapy with saline Group (*n* = 45)

The canals were irrigated with NaOCl at room temperature, followed by a final flush with cold saline [13]. The irrigation syringes in were kept in an ice box filled with cooling gel packs to maintain the liquid at a temperature of 2–4 °C. A thermocouple was used to confirm that the temperatures remained within the required range.

#### Group D Intra-canal cryotherapy with Hypochlorite group (*n* = 45)

The canals were irrigated with cold NaOCl and then rinsed with cold saline as a final flush [13]. The irrigation syringes in were kept in an ice box filled with cooling gel packs to maintain the liquid at a temperature of 2–4 °C. A thermocouple was used to confirm that the temperatures remained within the required range.

During canal instrumentation, irrigation was performed between rotary file sequences using a side-vented needle (30-gauge) positioned 2 mm short of the working length. Each interval was irrigated with 5 mL of 2.5% sodium hypochlorite (NaOCl). For the final flush, a saline solution was delivered over a 10-minute period using the same needle configuration to ensure thorough canal cleansing while maintaining consistent needle placement. Finally, the canals were dried with paper points corresponding to the master apical file, and the second apical fluid sample (S2) was collected.

### Obturation and postoperative instructions

Following radiographic confirmation of the master cone, root canal obturation was performed during the same visit using the cold lateral condensation technique. Gutta-percha points and a resin-based sealer (Meta BioMed, Korea) were utilized. Prior to obturation, the canals were irrigated with 17% EDTA to eliminate the smear layer. After completion, the access cavity was sealed with glass ionomer cement (Meta BioMed, Korea).

A postoperative radiograph was taken to verify the accuracy of the obturation. Patients were given detailed postoperative instructions and provided with a numeric Visual Analog Scale (VAS) chart to assess pain levels, ranging from 0 (no pain) to 10 (worst imaginable pain).

Patients were contacted by phone at 6, 12, 24, 48, and 72 h after the procedure to record their pain scores. Each patient was prescribed an analgesic containing 50 mg of diclofenac potassium (Cataflam^®^), with instructions to take one tablet every eight hours for three days if experiencing persistent or intolerable pain. Patients were also asked to document the number of analgesic doses taken during the follow-up period.

### Biochemical analysis

Substance P (SP) levels in samples S1 and S2 were measured using the Enzyme-Linked Immunosorbent Assay (ELISA). Split paper points were diluted in 600 mL of phosphate-buffered saline (PBS) at pH 7.4. The diluted samples were then centrifuged at 10,000 rpm for five minutes. SP concentrations were determined using a commercial ELISA kit (Catalog No. E-EL-0067, Elabscience Biotechnology), following the manufacturer’s protocol. The kit’s minimum detection threshold for SP was 3.9 pg/mL. Absorbance readings were obtained using a microplate reader (Spectra Max Plus 384, USA) at wavelengths between 420 and 450 nm. A standard curve was generated using known SP concentrations. The concentration of drug P in each sample was calculated based on this standard curve.

### Statistical analysis

Statistical analysis was performed with SPSS 27^®^, Graph Pad Prism^®^ and Microsoft Excel 2016. All data were explored for normality by using Shapiro Wilk Normality test Kolmogorov tests. Which revealed that all data deviated significantly from non-parametric distribution (*P* < 0.05), except age was normally distributed (*P* > 0.05). Comparison between different groups was performed by One Way ANOVA test in normal data. In non-parametric data, comparison was performed by using Kruskal Wallis test followed by Dunns test, comparison between different time points was performed by using Fridman’s test followed by Duns test, while comparison between before and after was performed by using Wilcoxon signed rank test. In categorical data (analgesics intake and sex) all comparisons were performed by using chi square test. The significant level was set to be at *P* ≤ 0.05.

## Results

**Fig. 1:**
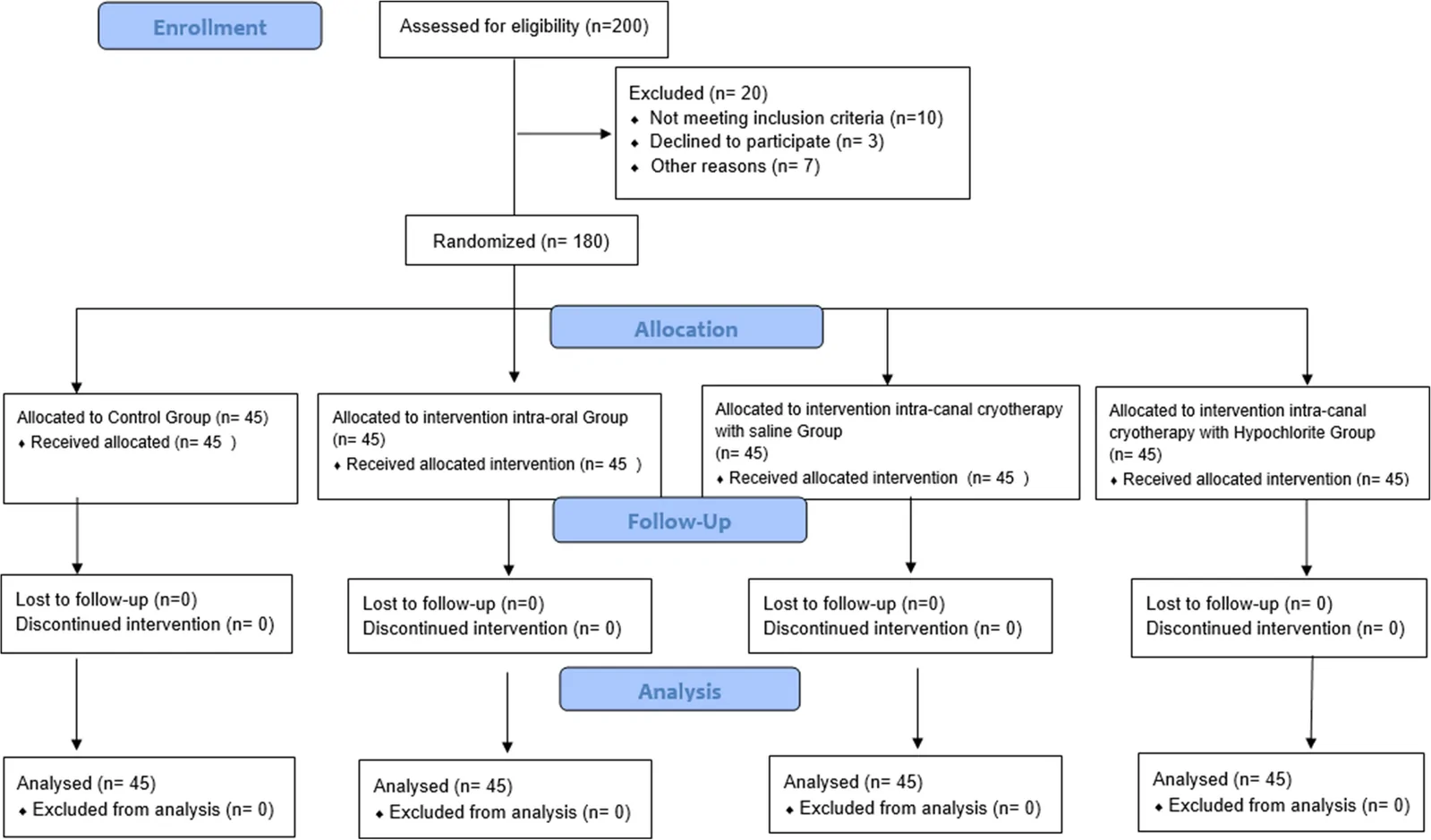
The consolidated standards of reporting trials flow diagram

The study enrolled 180 patients, with 45 individuals assigned to each group, in adherence to the principles of the Consolidated Standards of Reporting Trials (CONSORT) and the Declaration of Helsinki, as illustrated in Fig. 1.

The recall rate was 100%, with no exclusions or loss to follow-up. There were no significant differences among the groups regarding demographic characteristics (Table 1) or preoperative pain levels (Table 2).

**Table 1:** Comparison between groups regarding demographic data

		Group								*P* value
		Group A		Group B		Group C		Group D		
		Count	Column *N* %	Coun	Column *N* %	Coun	Column *N* %	Coun	Column *N* %	
Sex	male	21	46.7%	15	33.3%	17	37.8%	18	40.0%	0.67
	female	24	53.3%	30	66.7%	28	62.2%	27	60.0%	
Age		Mean	Standard Deviation	Mean	Standard Deviation	Mean	Standard Deviation	Mean	Standard Deviation	
		33.20	5.00	33.40	4.88	33.18	4.63	36.07	4.48	0.84

**Table 2:** Pain in all groups at different timepoints

Time interval	Group A		Group B		Group C		Group D		*P* value
	Min / Max (Median)	Mean ± SD	Min / Max (Median)	Mean ± SD	Min / Max (Median)	Mean ± SD	Min / Max (Median)	Mean ± SD	
6 h before	7.00 / 10.00 (8.00)	8.44 ± 0.97 aA	7.00 / 10.00 (8.00)	8.49 ± 1.1aA	7.00 / 10.00 (8.00)	8.18 ± 1.17 aA	7.00 / 10.00 (8.00)	8.44 ± 0.99 aA	0.34
6 h after	7.00 / 8.00 (7.00)	7.40 ± 0.50 aA	4.00 / 7.00 (5.00)	4.78 ± 0.85 bB	3.00 / 6.00 (5.00)	4.51 ± 1.08 bB	5.00 / 7.00 (6.00)	6.16 ± 0.88cB	< 0.0001*
24 h	5.00 / 6.00 (6.00)	5.67 ± 0.48 aB	2.00 / 5.00 (2.00)	2.56 ± 0.66 bC	0.00 / 3.00 (2.00)	1.98 ± 1.03 bC	1.00 / 2.00 (1.00)	1.24 ± 0.43cC	< 0.0001*
48 h	3.00 / 4.00 (4.00)	3.62 ± 0.49 aC	0.00 / 3.00 (1.00)	1.18 ± 0.83 bCD	0.00 / 2.00 (1.00)	0.80 ± 0.87 bcD	0.00 / 1.00 (0.00)	0.27 ± 0.45cD	< 0.0001*
72 h	2.00 / 3.00 (2.00)	2.47 ± 0.50 aD	0.00 / 2.00 (1.00)	0.56 ± 0.55 bD	0.00 / 1.00 (0.00)	0.09 ± 0.29 cD	0.00 / 0.00 (0.00)	0.00 ± 0.00cD	< 0.0001*
P value	< 0.0001*		< 0.0001*		< 0.0001*		< 0.0001*		

*Significant difference as *P* < 0.05

Means with different superscript letters (small per row /capital per column) were significantly different as *P* < 0.05

Pain intensity differed significantly among groups at all time intervals (*P* < 0.0001). At 6 h after treatment, Groups B and C showed significantly less pain scores than Group D, with no significant difference between them. Group A showed significantly highest pain scores (*P* < 0.0001). By, 24 h, Group D achieved the lowest pain scores followed by Group C and Group B and all Groups were significantly lower than Group A. At 48 and 72, the step wise reduction persisted with Group D consistently recording lowest pain levels and Group A maintain highest.

Regarding SP values, a significant difference in absolute SP reduction was observed among groups (*P* = 0.02). Group C demonstrated the greatest difference, while Groups B and D produced intermediate reductions without significant differences between them.

In terms of percentage change in SP, the difference among groups was highly statistically significant (*P* < 0.0001). Group C demonstrated the highest percentage of reduction and Group A showed the lowest percentage change, while Group D showed an intermediate reduction, not significantly different from either Group A and B. (Table 3)

**Table 3:** Differences and percentage of Reduction in SP

Parameter	Group A		Group B		Group C		Group D		*P* value
	Min / Max (Median)	Mean ± SD	Min / Max (Median)	Mean ± SD	Min / Max (Median)	Mean ± SD	Min / Max (Median)	Mean ± SD	
Difference	−3.24 / 343.38 (37.67)	63.68 ± 90.20 a	10.66 / 146.33 (37.33)	50.21 ± 39.60 ab	6.20 / 270.53 (82.72)	99.96 ± 85.62 b	0.59 / 238.13 (43.36)	52.90 ± 55.79 ab	0.02*
% of change	−1.40 / 58.90 (17.20)	19.43 ± 16.76 a	4.89 / 67.43 (24.60)	30.62 ± 18.63 b	14.70 / 74.20 (45.60)	44.27 ± 18.38 c	0.50 / 59.40 (24.70)	27.03 ± 17.29 ab	< 0.0001*

*Significant difference as *P* < 0.05

Means with different superscript letters small per row were significantly different as *P* < 0.05

**Fig. 2:**
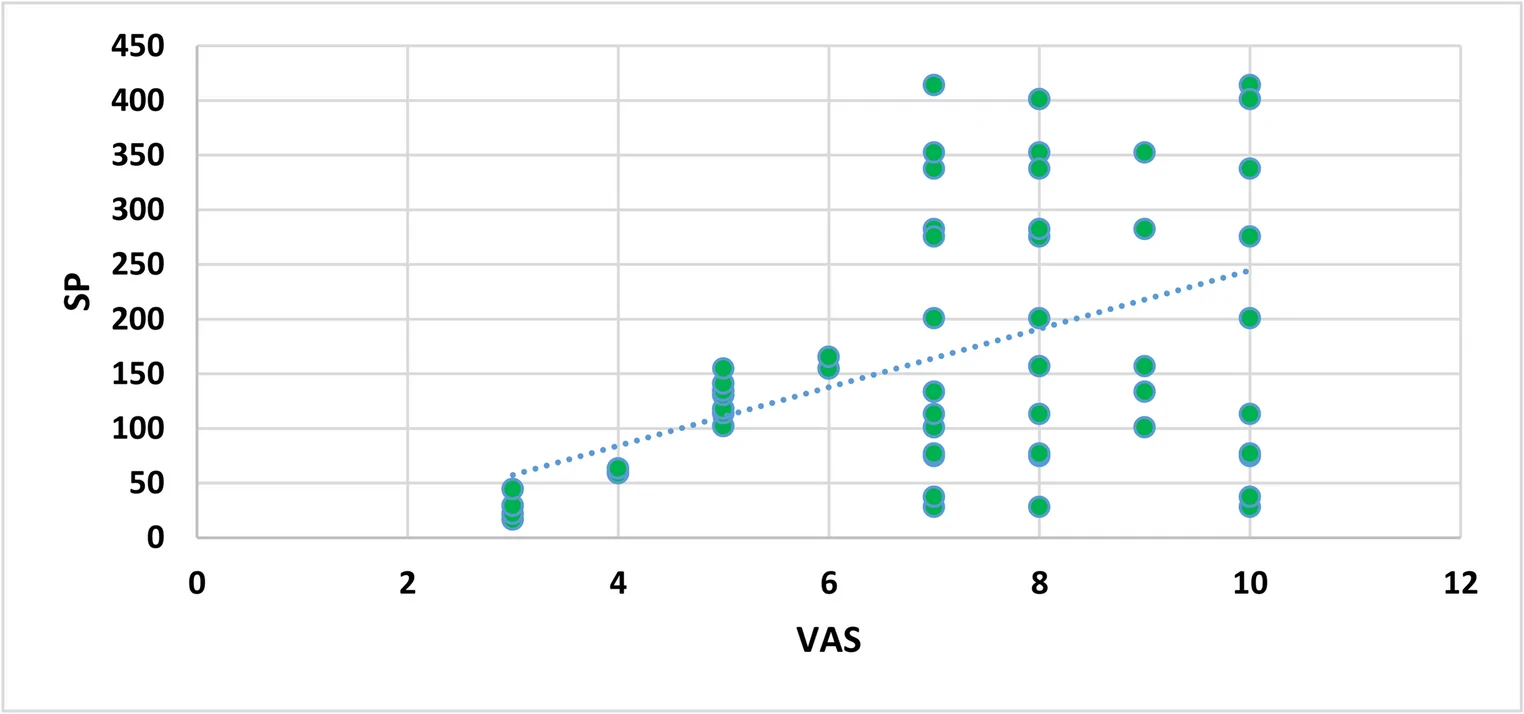
Scatter plot illustrating the correlation between VAS and SP

Using Pearsons correlation coefficient, the results demonstrated a moderate-to-strong positive correlation was observed between pain and SP (*r* = 0.52), which was statistically significant (*P* = 0.0001), This analysis was based on pain scores recorded 6 h after treatment, indicating a clear association between pain perception and SP vales. (Fig. 2)

## Discussion

Previous investigations have consistently demonstrated the effectiveness of cryotherapy in alleviating postoperative pain following endodontic treatment [7]. The available literature describes several cold application techniques, including intracanal cryotherapy [7, 14]—where cold saline is employed as a final irrigant—alongside intraoral methods [6] and extraoral applications [15]. Despite these promising approaches, the methodology and clinical effectiveness of cryotherapy remain non-standardized. In particular, there is no consensus regarding the optimal duration or mode of application, and the variability in reported protocols continues to hinder the establishment of a universally accepted guideline [13].

Accordingly, our study aimed to evaluate and compare the impact of using different cryotherapy techniques during endodontic treatment.

Preoperative pain is a strong predictor of postoperative discomfort [2]. To ensure the presence of inflammation, patients enrolled in this study presented with mandibular premolars diagnosed with irreversible pulpitis and symptomatic apical periodontitis. Pulp vitality was first confirmed by pain response to cold testing and later verified by bleeding during access preparation, which is considered the gold standard [16]. Apical periodontitis was diagnosed by percussion examination. Since only vital teeth were included, treatment was completed in a single session to avoid intracanal medicaments and exclude necrotic pulp cases. For obturation, the cold lateral condensation technique was selected over warm vertical compaction, as it is associated with reduced pain and patient discomfort, thereby minimizing bias in postoperative pain assessment [17].

As documented by Possoff A [18]., the effectiveness of cryotherapy is influenced by the thermal conductivity of the treated tissues. For this reason, intraoral cryotherapy was selected over extraoral application, as the latter is hindered by the insulating effect of muscles and adipose tissue in the cheeks, which limits cooling of deeper structures [19]. Moreover, intraoral application allowed us to shorten the duration of therapy to 30 min, thereby reducing the risk of potential complications such as nerve palsy [6].

Gel packs were preferred rather than ice packs due to their flexibility and better adaptation to the oral vestibule [6]. Merrick et al. [20] reported that while both cooled the skin within 10–15 min, gel packs maintained the lowered temperature for a longer duration. Since no clinical trials have compared intermittent versus continuous oral cryotherapy, this study adopted an intermittent protocol: the gel pack was applied for 10 min, removed for 2 min, and repeated twice, with a new pack used each time to ensure stable cooling.

In our study, sodium hypochlorite (NaOCl) was cooled to 2–4 °C for use as an irrigant. Previous research has shown that lowering the temperature does not significantly alter its antimicrobial properties [21]. This range was selected based on evidence that effective cryotherapy requires reducing tissue temperature to approximately 10 °C for 20 min to achieve anti-inflammatory benefits [22]. In endodontics, this has typically been accomplished either by irrigating with cold saline at 2.5 °C at the end of treatment or by using cold NaOCl throughout the chemo-mechanical preparation. Both approaches have been shown to lower the external root surface temperature to 10 °C, thereby producing the desired therapeutic effect [23].

Questionnaires and rating scales are frequently used in pain evaluation; one of the most popular approaches is the VAS [6]. These subjective metrics, however, might differ based on each person’s pain threshold [24]. Consequently, the use of objective biomarkers to supplement subjective evaluations has gained attention. Interleukin-6 (IL-6) and calcitonin gene-related peptide (CGRP) are two examples of inflammatory cytokines and neuropeptides that are known to be important in the pathophysiology of endodontic pain and inflammation [25].

The findings of our study showed no statistically significant differences between groups in terms of age, sex, or preoperative pain characteristics, suggesting that these variables did not influence the outcomes.

Our findings indicate that the use of cold NaOCl produced a statistically significant reduction in (POP). This effect was more pronounced in Group D (Intracanal cryotherapy with hypochlorite) than in the other cryotherapy techniques groups, which showed a significant decrease compared to the control. Across all groups, POP levels declined steadily throughout the observation period, consistent with previous reports [13, 26]. This outcome reflects both the effectiveness of root canal therapy in alleviating pain [27] and the additional benefits of cryotherapy, including lowering nociceptor activation thresholds [28], promoting vasoconstriction to limit inflammation [29], and reducing cellular metabolism to minimize tissue damage [29]. Extending the duration of cold irrigation further enhanced these therapeutic effects [13, 30].

The differences between the present findings and those reported in previous studies may be attributed to variations in study design, patient selection, and cryotherapy protocols. Unlike Gundogdu et al. [15] who evaluated mandibular molars and compared intraoral, extraoral, and intracanal cryotherapy using postoperative pain as the sole outcome, the present study included only mandibular premolars and incorporated both clinical (VAS) and biochemical (Substance P) assessments. Furthermore, Bazid and Kenawi [31] included cases with symptomatic irreversible pulpitis regardless of the presence of symptomatic apical periodontitis and evaluated only a single intracanal cryotherapy protocol using a final flush of cold saline. In contrast, the present study specifically enrolled patients with symptomatic apical periodontitis and compared three different cryotherapy protocols, allowing a more comprehensive evaluation of their clinical and biological effects. These methodological differences may explain the variation in the reported outcomes across studies and emphasize the importance of considering patient characteristics and treatment protocols when interpreting the effectiveness of cryotherapy.

The physiological effects of cold application are directly linked to its therapeutic benefits. Hemodynamically, cooling induces vasoconstriction, which reduces edema and inflammation by limiting leukocyte adhesion to the capillary endothelium and minimizing their migration [29]. At the cellular level, cold slows metabolic activity, thereby decreasing oxygen consumption and restricting tissue damage through its vasoconstrictive effect [29]. Neurologically, cryotherapy influences peripheral nerve endings by lowering nociceptor activation thresholds and reducing the conduction velocity of pain impulses—a phenomenon known as cold-induced neurapraxia [28].

Recent research has also highlighted the role of nerves in modulating inflammatory processes via neuropeptide release [32]. Substance P (SP), a neuropeptide secreted in response to noxious stimuli, initiates inflammation and significantly impacts immune responses and blood flow, playing a central role in conditions such as irreversible pulpitis [9, 33, 34]. In this study, SP levels were assessed by collecting apical fluid samples from the periapical area. This approach offered a distinct advantage, as it enabled evaluation of inflammatory mediator responses to chemo-mechanical procedures within a closed human environment, avoiding potential confounding factors associated with gingival crevicular fluid analysis [35]. Importantly, only patients who had not taken preoperative analgesics within seven days prior to treatment were included, thereby eliminating possible bias in SP expression [34].

The results of our study revealed that Group C (intracanal cryotherapy with saline) achieved the greatest reduction in (SP) levels, showing both a significantly higher percentage of reduction and the largest difference between pre- and post-treatment values. Group D (intracanal cryotherapy with hypochlorite) demonstrated an intermediate reduction, which was not statistically different from either Groups A(Control) and B (Intraoral cryotherapy).

A previous studies evaluating cold Irrigation and intraoral cryotherapy reported lower SP levels in the cryotherapy group compared to the control, though the difference did not reach statistical significance [6, 13]. Other investigations have focused on alternative inflammatory mediators, such as interleukin-6, and consistently found reduced levels in cryotherapy groups [25, 36].

The lower SP levels observed in Group C compared to Group D may be explained by several factors. Unlike NaOCl, saline is biologically inert and doesn’t introduce chemical irritation [37]. In contrast, NaOCl, despite being the irrigant of choice in endodontic practice [38], is known to exert cytotoxic effects on periapical tissues [37]. Furthermore, NaOCl’s tissue-dissolving reaction has potential exothermic nature, which may influence neuropeptide release and thereby affect SP levels [39]. In Group C, the final flush with cold saline likely induced a short-term thermal shock, producing rapid vasoconstriction followed by vasodilation [39]. This mechanism may be more effective in modulating nociceptor activity than the prolonged exposure to cold NaOCl in Group D, which tends to equilibrate with surrounding tissues over time [40]. Finally, it must be acknowledged that the VAS scale represents a subjective measure of pain intensity, influenced by individual pain thresholds [24]. For this reason, the integration of SP expression as biomarker provides an objective correlate to the clinical findings.

The relatively large standard deviations observed for the reduction in Substance P levels indicate considerable inter-individual variability in the inflammatory response [41]. Such variability is expected when evaluating neuropeptides, as their expression is influenced by patient-specific biological and immunological factors, including the baseline inflammatory status and host response to treatment [10]. Furthermore, a small number of participants exhibited minimal reductions or slight increases in SP levels following treatment, contributing to the wider dispersion observed, particularly in the control group. Despite this variability, the overall trends remained consistent across groups, and statistically significant differences were still detected, supporting the robustness of the study findings.

Our results demonstrated a moderate to strong positive correlation between Substance P (SP) levels and pain intensity, as reflected in VAS scores at 6 h post-treatment. This association underscores the pivotal role of SP in the pathophysiology of postoperative pain (POP) [42].

Although the study demonstrated statistically significant reduction in (POP) and SP levels, it is essential to consider whether these differences translate into meaningful improvements for patients. The observed reduction inVAS scores exceeded the commonly accepted threshold for the minimal clinically important difference (MCID), suggesting that patients would likely perceive a tangible improvement in their postoperative comfort. Similarly, the marked decrease in SP level supports a biologically relevant mechanism underlying pain modulation. Taken together, these findings indicate that cryotherapy techniques-particularly cold irrigants- are not only statistically valid but also clinically meaningful in enhancing patient outcomes. By rejecting the null hypothesis, our study confirms that cryotherapy provides significant therapeutic benefits in endodontics, functioning as an effective pain management strategy and contributing to improved patient outcomes.

Despite the confirmed results of the efficacy of different cryotherapy techniques in reducing (POP) and proinflammatory Substance P (SP) levels, certain limitations must be acknowledged. The primary limitation was the inability to blind the operator, as the use of cooled syringes and gel packs made the intervention evident. Also, we were unable to fully blind participants to the intervention, especially in the intraoral cryotherapy group, where patients could perceive the cold sensation. This may have introduced expectation-related bias, particularly given the subjective nature of VAS pain assessment. To mitigate this limitation, we included Substance P levels. Additionally, only one proinflammatory mediator was assessed, which restricts the scope of biological interpretation.

Despite these limitations, our findings support cryotherapy as a simple and effective adjunct for reducing POP in patient with symptomatic apical periodontitis. Future investigations should explore the long-term benefits of cryotherapy in endodontics and its broader impact on neuropeptides and other inflammatory mediators.

## Data Availability

No datasets were generated or analysed during the current study.
